# Enzymatic Digestion of Hyaluronan-Based Brain Extracellular Matrix *in vivo* Can Induce Seizures in Neonatal Mice

**DOI:** 10.3389/fnins.2019.01033

**Published:** 2019-09-26

**Authors:** Alena Balashova, Vladimir Pershin, Olga Zaborskaya, Natalia Tkachenko, Andrey Mironov, Evgeny Guryev, Leonid Kurbatov, Murat Gainullin, Irina Mukhina

**Affiliations:** ^1^Laboratory for Brain Extracellular Matrix Research, N. I. Lobachevsky State University of Nizhny Novgorod, Nizhny Novgorod, Russia; ^2^Cell Technology Group, Privolzhsky Research Medical University, Nizhny Novgorod, Russia; ^3^V. N. Orekhovich Institute of Biomedical Chemistry, Russian Academy of Medical Sciences, Moscow, Russia; ^4^Norwegian PSC Research Center and Institute of Clinical Medicine, Oslo University Hospital and University of Oslo, Oslo, Norway

**Keywords:** brain extracellular matrix, hyaluronic acid, seizure, behavioral tests, transcriptome analysis, neuroinflammation

## Abstract

It is well-known that hyaluronic acid (HA) as a component of brain extracellular matrix (ECM) plays a pivotal role in the nervous system and is involved in synaptic plasticity changes in vascular cognitive impairment and dementia. HA breakdown is a feature of the acute stage of stroke injury and may be detrimental through enhancement of the inflammatory response. Recent studies have shown that knockout mice lacking hyaluronic acid synthetase demonstrates epileptic phenotype *in vivo* and removal of HA leads to delayed development of epileptiform activity in cultured hippocampal neurons *in vitro*. Here, we studied whether digestion of hyaluronic acid in the hippocampus in early postnatal period can trigger seizures. Hyaluronidase (Hyal) (5 U/μl) was bilaterally injected into C57BL/6j mice (P17) CA1 field of hippocampus using the stereotaxic method to remove hyaluronan-based ECM. Transcriptome analysis of hippocampal tissue 2 h after enzymatic digestion of hyaluronan-based brain ECM revealed increased gene expression of proteins involved in inflammation reactions (TLR2, CCL2,3,5), neuroinflammation, axonal guidance and ephrin receptor signaling, versus the vehicle group. Mice injected with hyaluronidase exhibited delayed audiogenic seizures and improvement in working memory 72 h after injection, while there were no changes in locomotor activity, anxious level and exploratory behavior due to the open field test. The obtained results point to a link between the activation of neuroinflammation by enzymatic digestion of hyaluronan-based brain ECM during the neonatal period and their subsequent reactivity to seizures, which may play an important role in the functional features of the developing brain, including its seizure propensity.

## Introduction

Brain extracellular matrix (ECM) is a multicomponent complex. Members of the lectican family, chondroitin sulfate proteoglycans (CSPGs) such as neurocan, brevican, versican, and aggrecan with tenascins are anchored on hyaluronic acid (HA) backbone. HA is a unique non-sulfated glycosaminoglycan. It has a lot of physiological and biochemical properties including providing biomechanical integrity, altering tissue hydration and facilitating tissue assembly which are dependent on its size, concentration, and localization (George and Geller, 2018).

Hyaluronic acid is distributed throughout the ECM but the highest density of HA is detected around neuronal bodies and myelinated fibers (Perkins et al., 2017). ECM in the brain has two main states: (1) diffuse ECM, which is represented throughout the brain, and (2) densely packed matrix, forming so-called perineural networks (PNNs) (George and Geller, 2018). In the adult brain, PNNs generally surround parvalbumin-expressing GABA-ergic neurons by forming a lattice structure around synapses on the body and proximal dendrites, which may affect the development and stabilization of synapses in neonatal period (McRae et al., 2012). Moreover, depending on the proteoglycan component, PNNs prevent the formation of new synaptic connections by inhibiting axonal growth (Ueno et al., 2019). There is evidence that PNNs can also protect neurons from oxidative stress and glutamate-stimulated excitotoxicity (Ueno et al., 2019).

The formation of PNNs is a long activity-dependent process. *In vitro* studies have shown that formation of PNNs requires the activity of alpha-amino-3-hydroxy-5-methyl-4-isoxazole-propionic acid (AMPA) receptors and L-type voltage-dependent calcium channels (L-VDCC). During the period from the second to the third week after birth, the expression of crucial molecules of immature ECM such as tenascin-C and neurocan decreases, whereas expression of CSPGs such as aggrecan, brevican, versican V2, phosphacan, as well as tenascin-R and hyaluronan-binding protein Hapln1, are increased (Song and Dityatev, 2018). Rowlands et al. (2018) recently found that mice with knock-out of a gene responsible for aggrecan synthesis (*Acan*) has a significantly lower level of aggrecan, as well as phosphacan, neurocan, brevican, versican, and tenascin-R together with the reduced density of PNNs. This finding points to the crucial role of aggrecan in the formation of functional perineuronal nets.

Hyaluronic acid plays a pivotal role in synaptic plasticity during vascular cognitive impairment and dementia (Dityatev et al., 2009). Extracellular remodeling is a common response to different types of injuries and diseases. Upregulation of HA synthases (Has1 and 2) and hyaluronidases (Hyal1 and 2) in the cells from both stroke and peri-infarcted regions of the brain were demonstrated (Sherman et al., 2015). Recent studies have shown that knockout mice lacking hyaluronic acid synthase demonstrates epileptic phenotype (Arranz et al., 2014).

For most types of epilepsy, it is unknown how the hippocampus becomes hyperexcitable and how hyperexcitable cells integrate into a pathological circuit, resulting in the generation of spontaneous recurrent seizures. Subsequent transcriptional and translation alterations can induce changes in neuronal circuits, which predispose to development of seizures (Kim et al., 2017). Assessing the role of particular genes and pathways through the analysis of genome-wide gene expression is a promising tool to identify key trigger genes or pathways of epileptogenesis and other neurologic conditions (Younus and Reddy, 2017).

Previously, we have found that enzymatic digestion of hyaluronic acid by exogenous enzyme Hyal leads to the delayed development of epileptiform activity in cultured hippocampal neurons *in vitro* (Vedunova et al., 2013). In this study, we performed behavioral tests to identify seizure-like activity following Hyal intrahippocampal injections in C57BL/6j mice (P17). The ability for learning was assessed with a fear conditioning test. Open field exploratory test was used to estimate locomotor activity, anxiety and exploratory behavior. Transcriptome analysis of hippocampal tissue was carried out 2 h after enzymatic digestion of hyaluronan-based brain ECM to reveal changes in gene expression of different proteins. Immunohistochemical staining with anti-aggrecan antibodies was carried out to assess the integrity of PNNs.

## Materials and Methods

### Animals

Animals were housed in a specific pathogen-free animal facility at 20 ± 2°C with 60% humidity, a 12 h/12 h light/dark cycle, and *ad libitum* access to food and tap water until the surgery procedures. Animals were transferred into animal cages after surgery and maintained at the same environment with their male-littermates for 2–9 days. All experiments were carried out with an effort to minimize the number of animals used and the suffering caused by study procedures.

#### Ethics Statement

All animals were treated in strict accordance with ethical animal research standards defined by the Russian law and approved by the Ethical Committee on Animal Health and Care of Lobachevsky State University and Privolzhsky Research Medical University of the Nizhny Novgorod.

#### Hyaluronidase Injection

17-day-old C57BL/6J mice were divided among two groups: hyaluronidase-treated (Hyal) and phosphate-buffered saline (PBS) treated (Vehicle). Animals were deeply anesthetized with 1.5% isoflurane and the scalp was incised. For hyaluronidase injection, a 1 mm diameter portion of the skull was removed with a drill bit at stereotactic coordinates −2.06 mm anterio–posterior, 1.89 mm medio-lateral relative to bregma. 1.25 mm diameter glass capillary with open tip 5–10 μm diameter were used for injections. The glass capillary tip was placed 1.8 mm dorso-ventral to the skull surface. The speed of the liquid flow was maintained at 3–4 nl/s. The coordinates of the injection were calculated individually for each animal based on the ratio of the distance between bregma and lambda from those mentioned above. Animals in the experimental group (Hyal) were injected with 1 μl of Hyaluronidase from bovine testis (Sigma) diluted in 0.1 M phosphate buffered saline (PBS) at a concentration of 5 U/μl. Animals in vehicle group were injected with equal volume of 0.1 M PBS.

### Behavioral Tests

All the behavioral tests (*n* = 10/group) were carried out on second and third days after hyaluronidase injection.

#### Open Field Exploration Test

Mouse was placed in an open field chamber or arena (PanLab, Spain) and allowed to explore freely for 5 min. The locomotor activity (such as mean and max speed and traveled distance) in the whole arena was monitored and analyzed by an automated system (Smart v.3.0, PanLab, Spain). The distance traveled and the time spent in different parts of the arena (center and periphery) were estimated in a same way.

Anxiety (the duration of rearing and grooming in both compartments) was made in open field test.

#### Passive Avoidance Test

The short-term memory of experimental animals was evaluated by Passive Avoidance test (PanLab, Spain). The Passive Avoidance test is a behavioral model of conditioned reflex, consisting of the stages of training and reproduction of the reflex. The equipment was “Shuttle Box” divided into a dark and light compartment. Assessment of acquisition and retention of the passive avoidance task was carried out using identical illuminated and non-illuminated compartments containing a 50 W bulb. In brief, the animals underwent two separate trials: an acquisition trial and a retention trial, conducted 24 h after the acquisition trial. For the acquisition trial, the mouse was initially placed in the light compartment; the door between the two compartments was opened. When the mouse entered the dark compartment, the door automatically closed and an electrical foot shock (0.5 mA, 3 s) was delivered through the grid floor. The time before entering the dark room (latency) was registered. For the retention trial, the mice were placed in the light compartment as well as in acquisition trial and the time taken to enter the dark compartment was recorded again. If the mouse did not enter the dark compartment within 180 s, it was assumed that the mouse remembered the foot shock from the acquisition trial. The difference in time spent by an animal before entering dark chamber during retention and acquisition trials (delta time) was calculated.

#### Audiogenic Stimulus Assay

The animals were placed individually in the chamber (a glass cylinder 15 cm diameter, 25 cm height and video camera were placed a sound-proofing box) and were habituated for 5 min. Audiogenic stimulus was performed by a bell sound (100 dB) inside of box∼15 cm above the test subject. The stimulus lasted 60 s or until tonic-clonic seizure was observed visually. The behavior of the animal was recorded in three categories: wild running, tonic-clonic seizures, and death. If an animal displays the single wild run bout and stopped, thus displaying the low intensity fit score (Krushinsky–Molodkina, or KM, arbitrary unit “1”). The last stage of seizures goes through wild run which is followed first by clonic and then by tonic/clonic and tonic “full” seizure with extension of extremities and death (KM arbitrary unit “6”).

### Immunohistochemistry

#### Staining

The animals were transcardially perfused with 0.1 M PBS (pH = 7.4) at 2 h, 2 and 3 days after surgery, and subsequently followed by 4% paraformaldehyde (PFA) in PBS. Brains were then removed and fixed overnight in PFA, washed in PBS and dehydrated through a series of graded ethanol baths, and then infiltrated with paraffin; infiltrated tissues were embedded into paraffin blocks. Sections from paraffin blocks 5 um thick were made with semi-automated rotary microtome (Leica Biosystems, Germany). Every third slice was placed on an adhesive slide and dried. Paraffin sections were deparaffinized, rehydrated, treated with antigen retrieval buffer and washed in tris-buffered saline (TBS) (pH = 7.4). Permeabilization were made with 0.2% Triton X-100 (Sigma 93443-100ML) solution in TBS and was followed by blocking in 10% fetal cow serum (FCS), 1% bovine serum albumin (BSA) in TBS for 2 h. After that, sections were incubated overnight at 4°C with primary antibodies. The next day, sections were incubated with the appropriate species-specific fluoroconjugated secondary antibodies (Alexa Fluor 647 and Alexa Fluor 488; 1:400; Abcam) for 1 h. Primary antibodies used were rabbit polyclonal anti Aggrecan (ab36861; 1:100; Abcam), and chicken polyclonal anti NeuN as a neuronal marker (266006; 1:100; Synaptic Systems). The secondary antibody was goat polyclonal secondary antibody to rabbit IgG – H&L (Alexa Fluor^®^ 488) (ab150077; 1:400; Abcam) and goat polyclonal secondary antibody to chicken IgY – H&L (Alexa Fluor^®^ 647) (ab150171; 1:400; Abcam). Stained sections were mounted using Fluoroshield Mounting Medium with DAPI (ab104139, Abcam) and examined by fluorescence microscopy using a ZEISS LSM 880. Images were acquired controlling for equal exposure times using Zen 2.1 SP3 software. 1.2 Pl-Apo objective, pinhole 0.8 μm. Data from three channels (DAPI 405, Alexa Fluor 488, and Alexa Fluor 647) were collected by sequential scanning. Single confocal planes were analyzed to determine co-localization of signals in tissue sections.

We assessed the fluorescence intensity of anti-aggrecan antibody to assess the integrity of PNNs in four 212.5 × 212.5 μm fields of view from each slice. The average cell area and average area of extracellular space was also estimated using ImageJ. *N* = 3 animals/group, three slices from each animal.

### RNA Extraction

For RNA extractions, hippocampi were dissected on ice 2 h after injections and immediately frozen via liquid nitrogen with subsequent transfer into −80°C freezer.

Total RNA was extracted from hippocampi of animals from both Hyal and Vehicle groups. RNA extraction was performed with the RNeasy mini kit (Qiagen, Valencia, CA, United States). A NanoDrop^®^ NP-1000 spectrophotometer (NanoDrop Technologies, Wilmington, DE, United States) was used to measure the quantity of the RNA samples. The quality of the RNA samples was checked using a 2100 Bioanalyzer (Agilent Technologies Inc., Santa Clara, CA, United States), RNA integrity of each sample was assessed using ratio of 18S/28S RNA and RNA integrity number (RIN).

### Agilent Microarray Analysis

The synthesis of cDNA followed by the synthesis of cRNA with fluorescent labels was performed using the Agilent Low RNA Input Fluorescent Linear Amplification Kit (Agilent Technologies Inc., Santa Clara, CA, United States). 3-CTP or 5-CTP nucleotides with fluorescent labels (Agilent Technologies Inc., Santa Clara, CA, United States) were used for detection.

After determining the concentration of cRNA and evaluating the efficiency of fluorescent labels incorporating, the samples (750 ng each) were mixed in one tube and fragmented using reagents from the *in situ* Hybridization kit-plus kit (Agilent Technologies Inc., Santa Clara, CA, United States). After the end of fragmentation, 500 μl of the mixture was applied to Sure Print G3 Mouse Gene Expression v2 8 K× 60K Microarray Kit (Agilent Technologies Inc., Santa Clara, CA, United States) expression chips. These microchips contain 60,000 of 60 nucleotide probes immobilized on a glass and designed to analyze gene expression of the complete mouse genome (annotated genes with known and unknown function) in a given volume. Hybridization was performed in a rotary incubator (Agilent Technologies Inc., Santa Clara, CA, United States) at 65°C for 17 h. The chips were washed in an SSC buffer with the addition of Triton X102 according to the protocol of the manufacturer, then chips were scanned on a G2505B confocal laser scanner (Agilent) with a resolution of 10 μm. Extraction of DNA chip data and their primary processing were performed using the Feature Extraction 9.1 (Agilent Technologies Inc., Santa Clara, CA, United States) program.

The background signal was calculated and the fluorescence intensity in the red and green channels was calculated for each sample (spot). After determining the background signal level, all genes which are not expressed both in the Vehicle and Hyal samples were excluded from further analysis. Finally, the degree of difference in the signal of certain genes in different samples was evaluated (Vehicle, *n* = 4; Hyal, *n* = 4).

### Microarray Analysis

A microarray analysis was performed using BRB-ArrayTools^1^ to identify probe intensity values developed by Dr. Richard Simon and the BRB-ArrayTools Development Team that were significantly affected by hyaluronan digestion. Intra-array LOWESS normalizations were performed. The BRB-ArrayTools “Random Variance Model” option was used.

To identify the direct effect of hyaluronidase injection we compare Vehicle and Hyal groups. For this analysis two-sample *t*-test were used and *q*-value was 0.05 this resulted in 25 genes passed this filter (0.089%).

### Pathway Analysis

Ingenuity pathway analysis (IPA, Qiagen, Valencia, CA, United States) was used to perform “Core Analysis” to discover biological pathways that were significantly affected by hyaluronidase treatment. Data from microarray analysis were first uploaded into Qiagen’s IPA system for core analysis and then overlaid with the global molecular network in the Ingenuity Pathway Knowledge Base (IPKB).

Ingenuity pathway analysis was performed to identify canonical pathways, functions, upstream regulators that are most significant to microarray outcomes and to categorize differentially expressed genes in pathways and upstream regulators.

### Establishment of Protein–Protein Interaction Network

Search Tool for the Retrieval of Interacting Genes/Proteins (STRING) database^2^ was used to analyze protein interactions (Szklarczyk et al., 2014). We modeled a network of 22 genes where the interaction scores above 0.4 was considered as *de facto* interaction with the high confidence (≥0.700). After modeling the interactions network, we performed the *k*-means clustering (number of clusters = 3).

### Statistical Analysis

All data quantification is presented as the mean ± standard error of the mean (SEM). Statistical analysis was performed using GraphPad Prism 6.0. Normality test was performed using Shapiro–Wilk criterion. The Mann–Whitney test and 2-way ANOVA multiple comparison test with Bonferroni’s correction were used to quantify behavioral test results. The difference between groups was considered significant if the *p* value was less than 0.05.

## Results

### Locomotor Activity, Anxiety, and Exploratory Behavior

**FIGURE 1:**
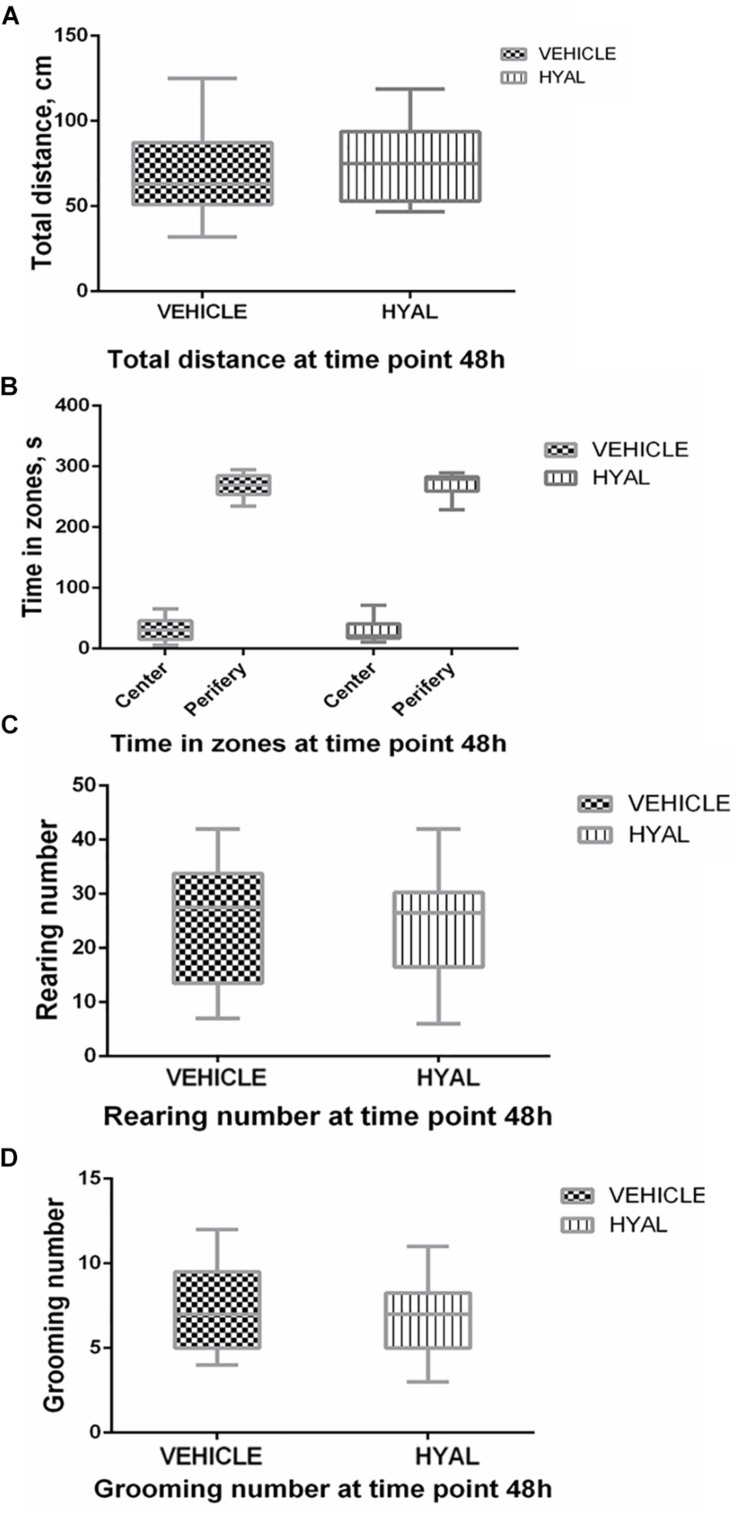
Open field test results at the time point 48 h. Hyal animals in Open field test show neither increase nor decrease in anxiety and exploratory behavior versus vehicle group on time points of 48 h. There was no difference in locomotor activity of animals as indicated by traveled distance **(A)**. Time spent by animals in center and periphery of Open field didn’t differ as well **(B)**. Anxiety level of Hyal animals didn’t differ refer to Vehicle group as was shown by rearing and grooming analysis **(C,D)**. Figures show mean ± SEM, *N* = 10 per group. Two-way ANOVA and Mann–Whitney test didn’t reveal any significant difference (*p* > 0.05).

**FIGURE 2:**
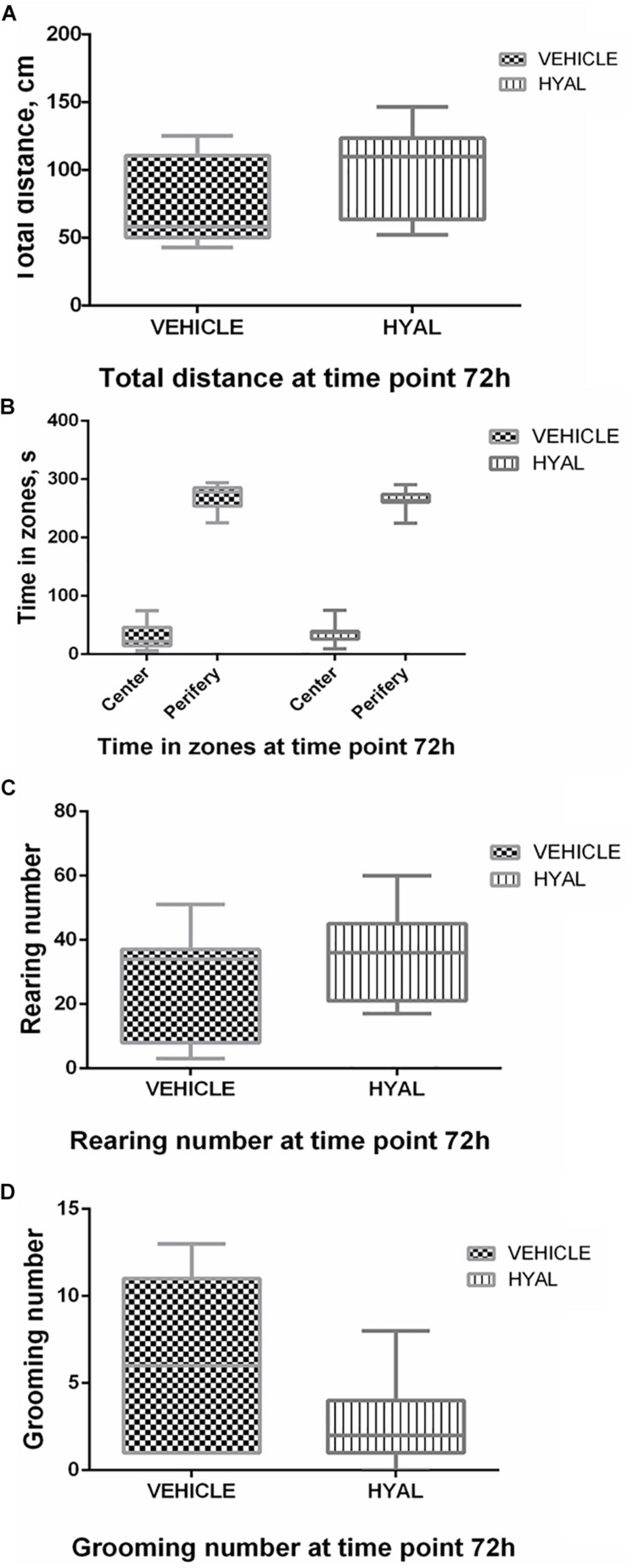
Open field test results at the time point 72 h. There was no difference in locomotor activity of animals as was shown by traveled distance **(A)**. Hyal animals exhibited normal aversion to brightly lit open areas as well as Vehicle animals did, as was shown by time spent by animals in center and periphery of Open field **(B)**. Anxiety level of Hyal animals didn’t differ refer to Vehicle group as was shown by rearing and grooming analysis **(C,D)**. Figures show mean ± SEM, *p* < 0.05. *N* = 10 per group. Two-way ANOVA and Mann–Whitney test didn’t reveal any significant difference.

Hyal animals in Open field test were shown neither increase nor decrease in anxiety and exploratory behavior versus vehicle group on time points 2 and 3 days. Locomotor activity wasn’t changed after hyaluronidase injection as well (Figures 1, 2).

### Short-Termed Memory Changes

**FIGURE 3:**
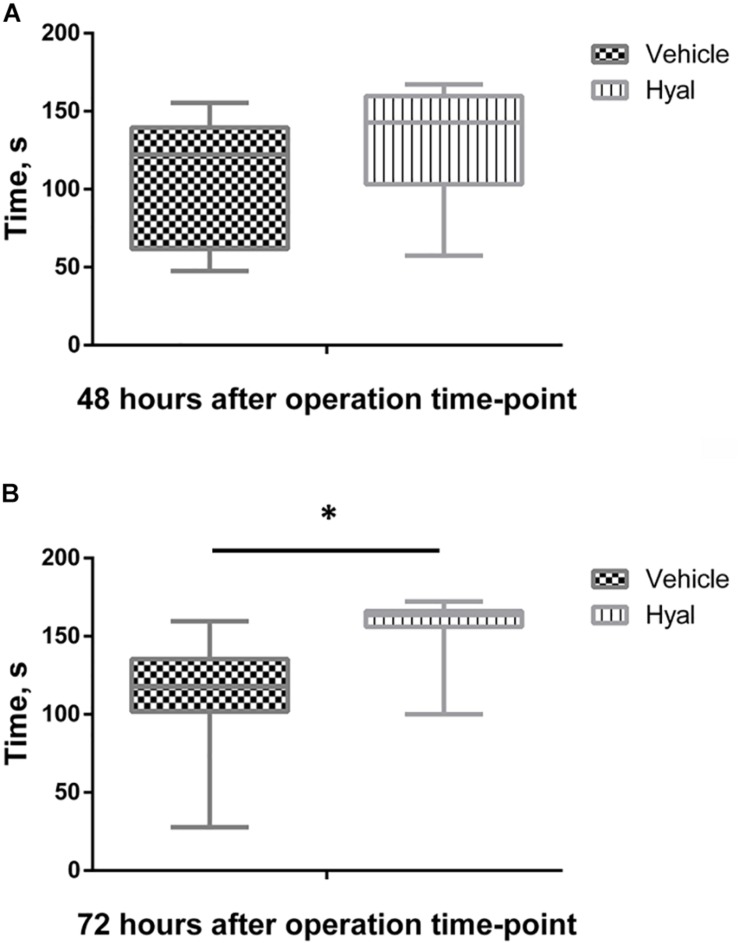
Passive avoidance test results. Time before enter dark compartment in acquisition and retention trials at time points of 48 h **(A)** and 72 h **(B)**. *Y* axis indicates the difference between latency time before entering the dark room in acquisition and retention sessions (delta time). All the animals entering dark compartment during acquisition trial. At the time point of 48 h after hyaluronidase injection animals from Hyal group remembered foot shock in 90% cases, while animals from Vehicle group remembered aversive stimuli only in 60% cases, but Mann–Whitney test showed no significant difference. At the time point of 72 h 100% of animals in Hyal group avoided entering the dark compartment during retention trial versus 70% of animals in Vehicle group, delta time in two groups significantly differs. Figures show mean ± SEM, *N* = 10 per group, ^∗^*p* < 0.05.

At the following time point: 48 h after hyaluronidase injection, animals from the Hyal group remembered foot shock in 90% of cases, while animals from Vehicle group remembered aversive stimuli only in 60% of cases. However, the difference between latency time before entering the dark room in acquisition and retention sessions (delta time) in two groups were not significantly different. 100% of animals in Hyal group avoided entering the dark chamber during retention trial versus 70% of animals in Vehicle group, delta time were significantly higher in Hyal group (Mann–Whitney test, *p* < 0.05). Delta time for both time points is shown in Figure 3.

### Audiogenic Seizures

It was shown that hyaluronidase injection can promote audiogenic seizures in neonatal animals. 20% of animals in Hyal group demonstrated wild running, 10% showed nearly complete tonic extension except hind feet (KM arbitrary unit “4”), and 10% of animals underwent all the stages of KM scale and died (KM arbitrary unit “6”) after audiogenic stimuli at the time point of 48 h. In the vehicle group, 10% of animals demonstrated wild running during stimulation.

**FIGURE 4:**
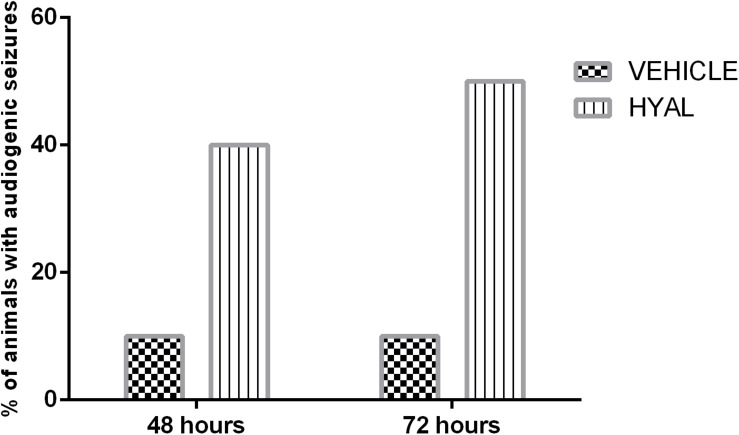
Audiogenic seizures test results. Data shown in the diagram represents the percent of animals demonstrating audiogenic seizures. *N* = 10. The percent of animals in Vehicle group demonstrating wild running during audiogenic stimuli stayed at the level of 10% on the time points of 48 and 72 h. 40% of animals in Hyal group in total demonstrated audiogenic seizures at the time point of 48 h. 20% of animals demonstrated wild running, 10% showed nearly complete tonic extension except hind feet (KM arbitrary unit “4”) and 10% of animals underwent all the stages of KM scale and died (KM arbitrary unit “6”) after audiogenic stimuli at the time point of 48 h. At the time point of 72 h in vehicle group number of animals with audiogenic seizure was even higher – 50% in total. 40% of animals demonstrated wild running and 10% of animals demonstrated seizures of KM arbitrary unit “4.”

The number of animals with audiogenic seizures was even higher at the time point of 72 h in the Vehicle group; 40% of animals demonstrated wild running and 10% of animals demonstrated seizures of KM arbitrary unit “4,” while in Vehicle group situation stayed the same (Figure 4).

### PNN Immunostaining

**FIGURE 5:**
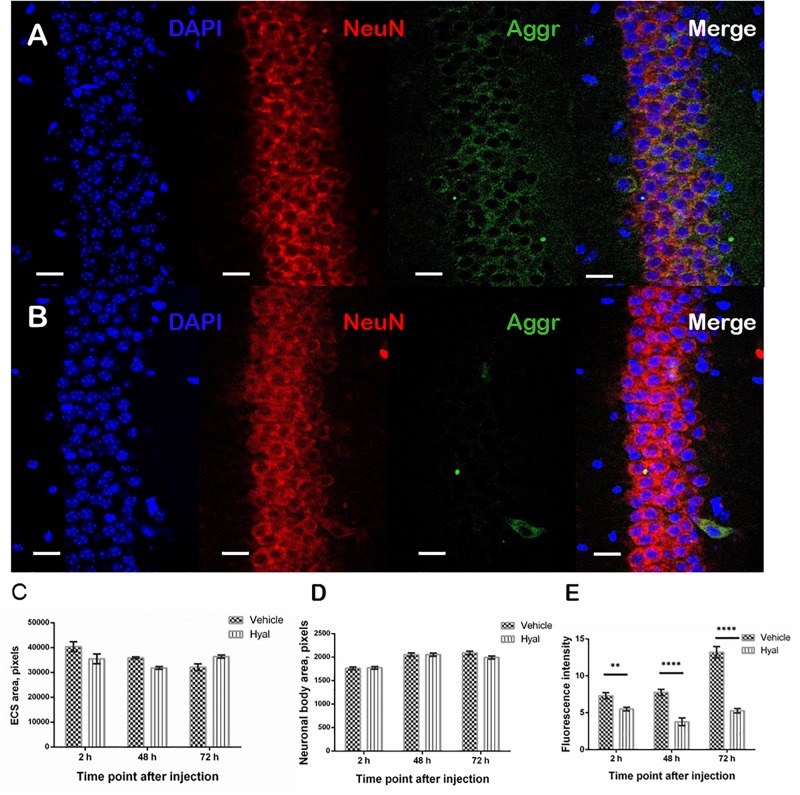
Immunohistochemical staining of paraffin embedded brain slices. Images obtained by fluorescence microscopy at the 48 h after injection time point from Vehicle **(A)** and Hyal **(B)** animals brain slices. The nuclear marker DAPI is shown in blue. Aggrecan, the component of perineuronal nets, is shown in green, neuronal marker NeuN is shuwn in red. The scale is 20 μm. We measured ECS area **(C)** and average neuronal body area **(D)** at the 2, 48, and 72 h after injection time points. Mann–Whitney test didn’t reveal any differences. The presence of aggrecan was measured by fluorescence intensity of green channel **(E)**. There is significant decrease in aggrecan component at all the time points but the minimum was fixed 48 h after injections. At the time point of 72 h the level of aggrecan starts to restore. ^∗∗^*p* < 0.005, ^****^p < 0.0001.

It was shown that, 2 h after hyaluronidase injection, there was no detectable aggrecan agglomerates surrounding pyramidal neurons in hippocampal regions of injections in Hyal group in comparison with the Vehicle group. The trend persisted in both Hyal and Vehicle groups 48 and 72 h after injection (Figures 5A,B). The analysis of the area of extracellular space and an average cell area didn’t reveal any significant differences between Hyal and Vehicle groups (Figures 5C,D). Fluorescence intensity analysis showed that the level of aggrecan in Hyal group reached its minimum at the 48 h after injection (Figure 5E) and started to restore at 72 h time point.

### Analysis of Differential Expressed Genes and Their Interactions

**FIGURE 6:**
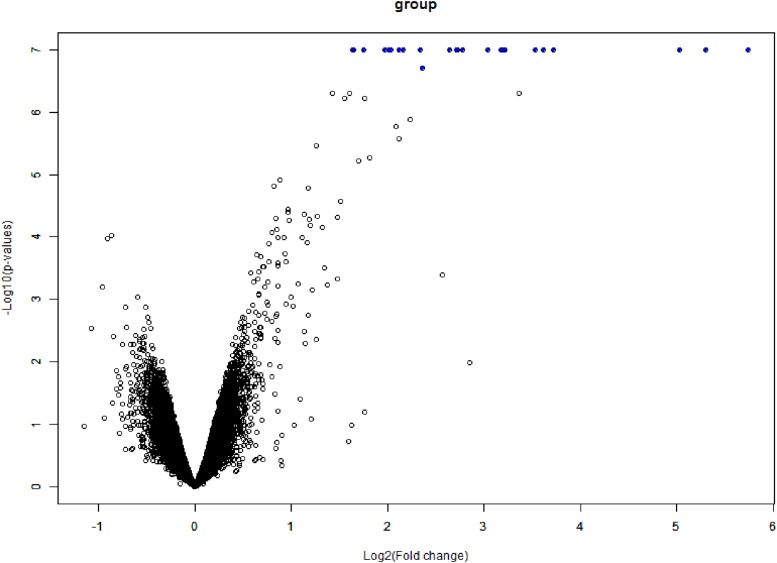
Volcano plot of DEGs. The *x*-axis is logFC and the *y*-axis represents –log10 (adjusted *P*-value). The blue dots represent the DEGs, while the black dots represent genes that were not differentially expressed. DEGs, differentially expressed genes; FC, fold change.

The data expression matrix was processed with BRB-array tools, whole gene expression data presented in Figure 6.

**FIGURE 7:**
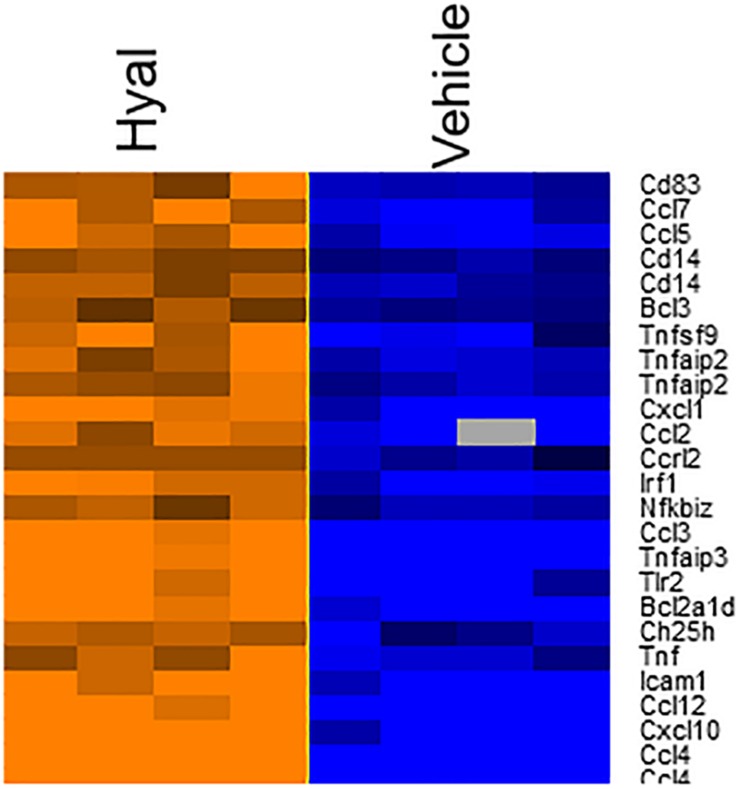
Heatmap of genes involved in response to hyaluronan digestion. The expression levels of genes were indicated by the color bar above the heatmap. Orange color indicates the increased expression whereas blue color indicates the decreased expression. Data log_2_ normalized.

According to the heat map (Figure 7), hyaluronidase treatment resulted in strong difference in comparison to vehicle control (PBS). The total number of DEGs were 25 (FDR = 0.05, *P* value ≤ 0.05, Benjamini–Hochberg correction).

**FIGURE 8:**
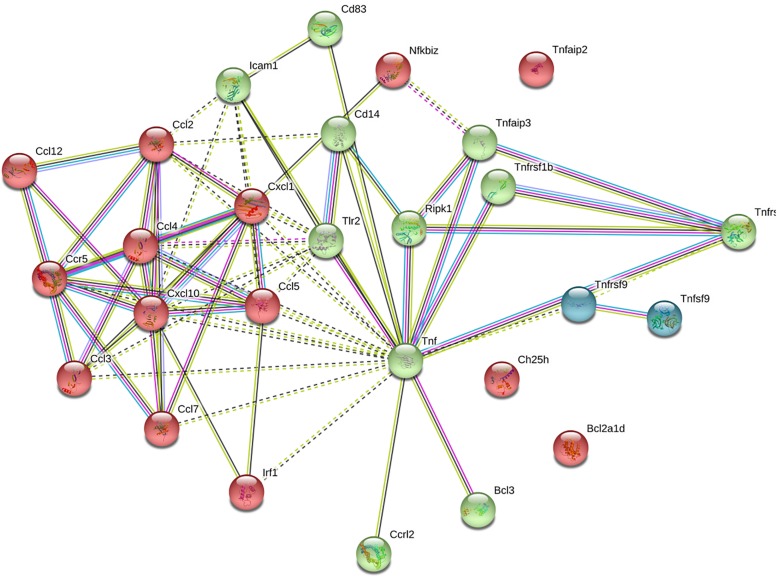
Protein–protein interaction networks of differentially expressed genes upon hyaluronidase treatment. Using STRING database 25 genes were used to construct the PPI network. PPI, protein–protein interactions. Colors of nodes indicate result of clustering. Nodes represent proteins, edges represent protein–protein associations. Dotted lines indicate insignificant link, solid lines indicate significant interactio, pink lines indicate experimentally determined interaction, blue lines from curated database, black lines indicate co-expression, yellow lines indicate text mining, and gray lines indicate protein homology.

Data from the STRING database revealed that several of the genes interacted with each other. In total, 25 genes were used to form the network complex (Figure 8). The network contained 27 nodes and 70 edges. Among them, seven nodes were the most significant (node degree ≥ 5). These were CCL2, CCL4, CCL5, CXCL1, CXCL10, CCR5, and TNF. Among seven nodes, TNF exhibited the highest node degree (*n* = 10).

### Functional Analysis

**FIGURE 9:**
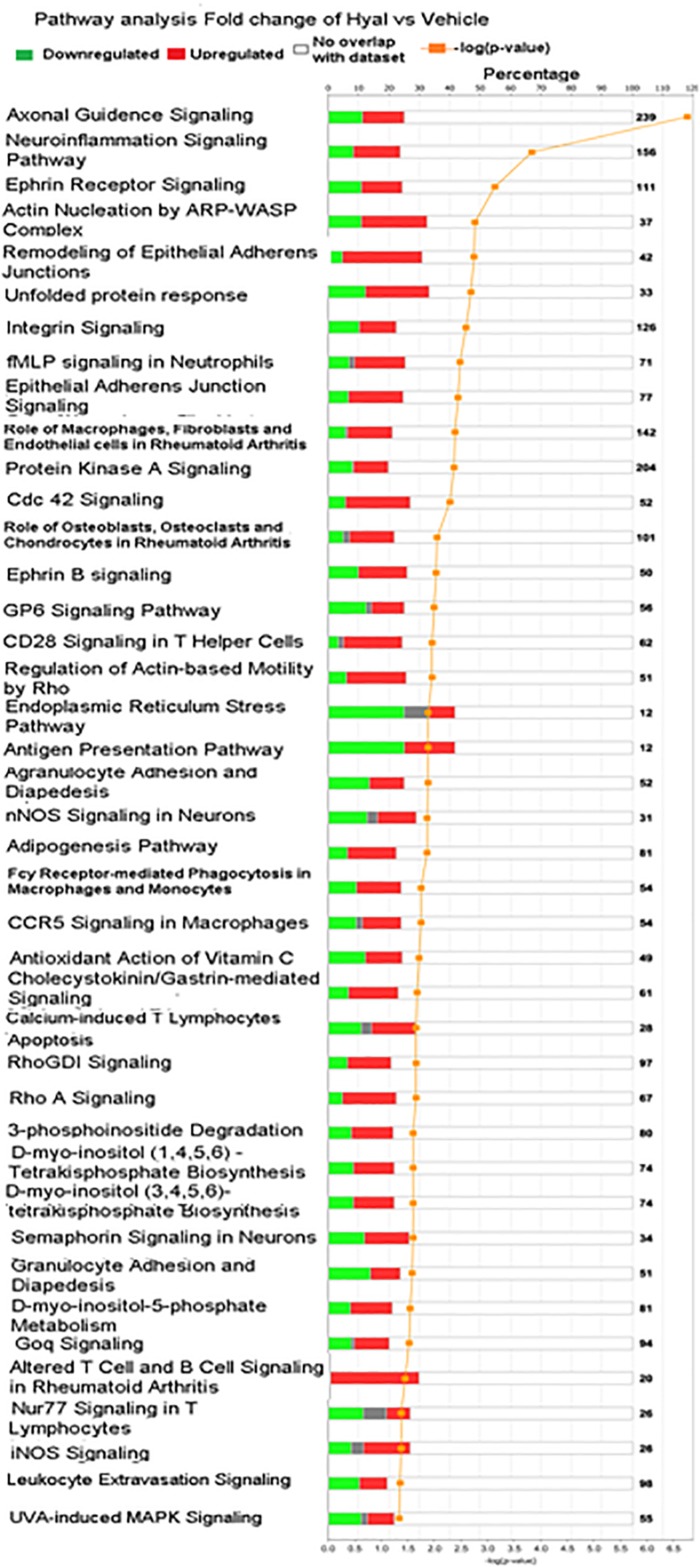
Significantly enriched pathways of significant genes in response to hyaluronan digestion. Green bars represent number of downregulated genes, red bars represent number of upregulated genes, gray bars represent no change of expression, orange curve with dots represent (–log_10_ (*P* value) – FDR), numbers in right border reflect number of genes including in canonical pathway.

To obtain an elementary investigation of the molecular mechanisms underlying disruption of hyaluronan matrix, microarray data were submitted to IPA core analysis. The differentially expressed genes were categorized to related canonical pathways based on IPKB. The top enriched categories of canonical pathways with *P* value < 0.05 are listed in Figure 9. Among pathways with −log_10_ *P* value, 3 include the Axonal guidence signaling, Neuroinflammation signaling pathway and Ephrin signaling pathway (Yu et al., 2016).

As hyaluronidase-treatment changed the profile of DEGs and signaling pathways, upstream regulators can also be changed. To investigate this issue, we tested DEGs identified upon Hyal treatment for enrichment of known key transcriptional regulators using the Upstream Regulator Analysis within the IPA Knowledgebase contains information about known regulators targets and can predict which transcriptional regulators are responsible for certain biological processes or it can suggest pathways and the potential impact regulators may have on those pathways.

**FIGURE 10:**
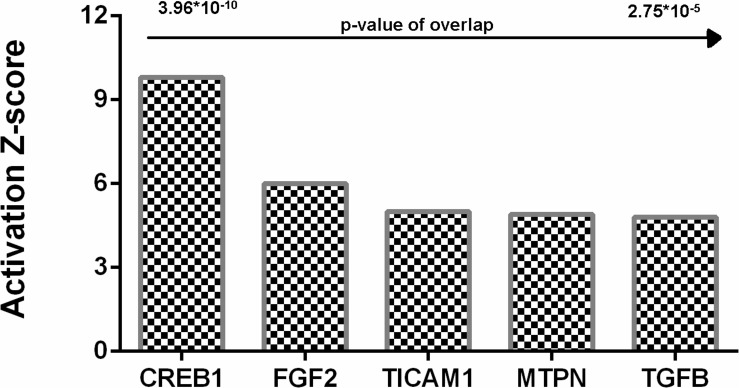
Upstream regulators identified by Ingenuity Pathway analysis for hyaluronidase dependent treatment. Positive activation *Z*-score (>2) indicates activation of these upstream regulators. CREB1, cAMP response element-binding protein; FGF2, fibroblast growth factor 2; TICAM1, TIR domain-containing adaptor molecule 1; MTPN, myotrophin; TGFB, Transforming growth factor beta.

Analysis identified five genes upregulated in hyaluronidase-treated hippocampal tissues (Figure 10) with a significant number of their targets (*p* < 10^–5^) changed in a direction which indicates an activated regulator status on a protein level (positive *Z*-score > 2).

## Discussion

Epilepsy is a most common neurological disorder all around the world. There are a lot of people suffering secondary epilepsy after brain infections or non-infectious (sterile) inflammatory response, traumatic brain injury or brain tumors (for review see Lowenstein, 2009; Vezzani et al., 2016). There is a large amount of mechanisms leading to seizure appearance and lots of it needs further investigation (Klein et al., 2018). The reasons for seizure occurrence in early postnatal development is the less studied subject in the field.

Brain ECM was shown to affect the maturation of central nervous system during early developmental stages (McRae et al., 2012; Ueno et al., 2019). We assumed that the destruction of ECM molecules during the early postnatal period may lead to the occurrence of some pathological states. Indeed, it was shown that 40 and 50% of animals after bilateral intrahippocampal hyaluronidase injections at the 48 and 72 h time points, respectively, exhibited seizures. The mechanism of epileptogenesis after ECM digestion during early development is not known so we decided to make a full transcriptomic analysis to unveil the possible pathways and detect target molecules for a further correction. Our transcriptomic analysis with further analysis of interacting genes/proteins revealed activation of a number of genes implicated in inflammatory responses (as far as cytokine-cytokine receptor interaction pathway was activated) in the Hyal group. Enzymatic digestion of hyaluronan produces a lot of low molecular weight fragments (<500 kDa), which can trigger an inflammatory response. During the inflammation, chemokines such as CCL2, CCL3, and CCL 5 can activate pathways leading to status epilepticus (Arisi et al., 2015; Cerri et al., 2016; Tian et al., 2017). In the current study, transcriptomic analysis showed the activation of the chemokine pathway due to upregulation of TLR2. Activation of TLR2 by short fragments of hyaluronan and disruption of CD44 can induce an inflammatory response (Jiang et al., 2011). Signals from these receptors can be transmitted through NF-κB transcription factor upregulating the expression of chemokines (CCL2,3,4,5,7,10,12, CXCL 1,10), which can act through ERK_1__,__2_ enhancing the production of TNF and activating CREB1 expression (Bose and Cho, 2013; Cekanaviciute and Buckwalter, 2016). Activation of CREB and glial-mediated production of TNF-α may led to enhancing the release of glutamate. Imbalance of glutamate production can cause excitotoxicity and seizures (Li and Ransohoff, 2008; Vezzani et al., 2016). These findings coincide with the opinion that at least 40% of all epilepsies have structural or metabolic causes as a consequence of diverse brain injuries and the most common risk is inflammation during infections and sterile inflammation during stroke and autoimmune encephalitis (Vezzani et al., 2016).

However, animals in the Hyal group didn’t show any differences in locomotor activity and anxiety level. This finding correlates with the previously made by Hosseini et al. (2018) where the overexpression of inflammatory factors didn’t affect the activity of animals in open field test, although the chronic inflammation can lead to a memory loss (Zhou et al., 2016). In contrast, we demonstrate that hyaluronidase-treated animals showed elicited ability for learning. At the time point of 48 h after surgery, 90% of animals in Hyal group learnt aversive stimuli versus 60% of animals in Vehicle group. The percentage of animals that learnt the task 72 h after surgery was 100 and 70, respectively, although Mann–Whitney test revealed the significant difference in delta time only at the 72 h time point. According to transcriptome analysis, this memory improvement might be due to an activation of *BCL3* and *IRF1* genes directly interacting with CREB, which is tightly connected with long-term potentiation and memory (Silva et al., 1998). Facilitation of working memory can also be explained by the fact that ECM modification leads to the formation of a new structural and functional connections between neurons, changes the ratio of inhibitory and excitatory neurons leading to changes of plasticity (Morellini et al., 2010). It had also been shown that some matrix proteins have a particular importance for the behavior, spatial, and contextual memory. For example, decreased expression of reelin is a common characteristic among neurological and mental disorders such as autism, schizophrenia, depression and bipolar disorder (Ferrer-Ferrer and Dityatev, 2018), and knock-out mice lacking aggrecan demonstrated improvement in memory in spontaneous object recognition test (Rowlands et al., 2018).

There are pathways activated after several types of cerebral insults, which are associated with epileptogenesis or epilepsy progression such as Janus Kinase/Signal Transducer and Activator of Transcription (JAK/STAT) and mammalian Target of Rapamycin Complex (mTORC) (Klein et al., 2018). These signaling pathways mediate cellular physiological mechanisms that are critical for memory formation, long-term depression (LTD) and long-term potentiation (LTP). The JAK/STAT pathway is activated after many types of epileptogenic injuries in experimental models, including TBI, SE and stroke (Satriotomo et al., 2006; Lund et al., 2008; Zhao et al., 2011). It regulates expression of genes critical for multiple essential functions including cell proliferation, differentiation, neurogenesis, learning and memory. We found the elevation in *NFKBIZ*, *IRF1*, and *BCL3* genes, which tightly interact with members of the JAK/STAT pathway. Structural or dysplastic lesions associated with infantile spasms in rodents or humans also demonstrate overactivation of the mTORC1 pathway in the epileptogenic area (Galanopoulou et al., 2012). We found an upregulation of TNF, which is involved in this pathway and TLR2, TNFAIP3, and CD14, which directly interact with other members of mTORC1 signaling pathways.

Brain tumors are the other pathological process that can cause seizures. One of the factors which contribute to peritumoral seizures is glioma-released glutamate, which presumably acts through chronic overactivation of neuronal glutamate receptors enhancing the excitatory drive in the cortex (Buckingham et al., 2011; Yuen et al., 2012). A recent study demonstrates that seizures can appear as a result of the loss of fast-spiking interneurons concomitant with a significantly reduced firing rate of those that remain due to the degradation of PNNs that surround fast-spiking interneurons, resulting in alterations of its membrane capacities (Tewari et al., 2018). It also should be noted that treatment with a GM6001, a broad-spectrum inhibitor of MMPs, leads to retention of the PNN’s structure and, most importantly, membrane properties of FSNs in GM6001-treated mice were essentially identical to the control group. The alteration of membrane capacities may also contribute to epileptogenesis after hyaluronidase injections.

There is an idea that seizures appearing in mice with ECM deficiency are linked with the reduction of ECS (Arranz et al., 2014). However, our immunohistochemical analysis didn’t reveal any significant differences in ECS area and in average neuronal body area at all time points, which means that seizures appeared after digestion of HA during postnatal stages were not a result of changes in osmotic parameters of cell environment.

Brain ECM is known to play a pivotal role in the functioning of the nervous system, especially during ontogenesis, but some of mechanisms by which it regulates neuronal activity are still hidden from the scientist’s eye. Therefore, our study provides new insight into the mechanisms of epilepsy development in early postnatal period, showing the role of hyaluronic acid in the stabilization of morphology and neural network activity. These mechanisms can become potential targets for the correction of seizures during early postnatal development. Proteomic analysis is needed to reveal the precise mechanism of epileptogenesis in neonatal animals after ECM digestion. It also remains unknown how long the hyperexcitable state of the brain is preserved after hyaluronan digestion.

## Data Availability Statement

The datasets generated for this study are available on request to the corresponding author.

## Ethics Statement

The animal study was reviewed and approved by the Ethical Committee on Animal Health and Care of N. I. Lobachevsky State University of Nizhny Novgorod and Privolzhsky Research Medical University, Nizhny Novgorod.

## Author Contributions

IM initiated the study. AB, VP, and MG generated a detailed plan of the experiments and supervised the research. AB, OZ, NT, and AM performed the behavioral and immunocytochemistry experiments. VP, EG, LK, and MG performed the transcriptome analysis. AB, VP, MG, and IM analyzed the data and wrote the first draft. All authors edited the manuscript.

## Conflict of Interest

The authors declare that the research was conducted in the absence of any commercial or financial relationships that could be construed as a potential conflict of interest.

## Footnotes

1 http://brb.nci.nih.gov/BRB-ArrayTools

2 http://string-db.org/
